# Isolated White Lupin Proteins Beneficially Modulate the Intestinal Microbiota Composition in Rats

**DOI:** 10.3390/nu17030551

**Published:** 2025-01-31

**Authors:** Luis A. Rubio, Giulia Chiesa

**Affiliations:** 1 Department of Animal Nutrition and Sustainable Production, Estación Experimental del Zaidin (CSIC), Profesor Albareda 1, 18008 Granada, Spain; 2 Department of Pharmacological and Biomolecular Sciences “Rodolfo Paoletti”, Università degli Studi di Milano, Via Balzaretti 9, 20133 Milan, Italy; giulia.chiesa@unimi.it

**Keywords:** casein, intestinal microbiota composition, lactalbumin, *Lupinus albus*, protein isolates, rat

## Abstract

Background: Previous work has shown that the mostly beneficial modulation of intestinal microbiota generally found with legume-based diets is likely to be due, at least in part, to their constituent protein components. Objectives: The faecal microbiota composition was studied in rats fed diets differing only in their constituent proteins. Methods: Rats (*n* = 10/group) were fed for 28 days diets based in milk [(lactalbumin (LA), casein (CAS)], or white lupin (*Lupinus albus*) protein isolate (LPI). Results: Significant differences among the three groups in bacteria composition and functionality were found by both qPCR and Illumina sequencing analysis. Significant (*p* < 0.01) differences were found by ANOSIM and Discriminant Analysis among groups at the family, genus and species levels in both microbiota composition and functionality. A number of groups able to explain the differences between animal (casein, lactalbumin) and lupin proteins were revealed by LEfSe and PCA analysis. Specifically, feeding the CAS diet resulted in lower *Bifidobacteria* and *Lactobacilli* compared to the other diets, and the LPI diet gave place to lower *Enterobacteria* than CAS, and lower *Escherichia*/*Shigella* than LA and CAS. Differences in the LA group were attributable to *Bifidobacterium* spp., *Collinsella* spp. (in particular *C. stercoris*), *Bacteroides* spp., *Eubacterium* spp. (in particular *E. dolichum*), *Roseburia* spp. (in particular *R. faecis*), and *Oscillospira* spp. In the case of the CAS group, the organisms were *Parabacteroides* spp., *Blautia* spp., *Enterobacteriaceae* spp., *Turicibacter* spp., species from *Christenellaceae*, species from *Alphaproteobacteria* and *Mogibacteriaceae*, *Coprobacillus* spp. and *Dorea* spp. In the case of the LPI group, the organisms were *Lactobacillus* spp. (*Lactobacillus* spp. and *L. reuteri*), species from *Clostridiaceae*, species from *Peptostreptococcaceae*, species from *Erysipelotrichaceae*, and *Adlercreutzia* spp. Conclusions: Based on the results obtained, LPI is likely to beneficially modulate the intestinal microbiota composition in rats. Additionally, LA-based diet was associated to a healthier microbiota composition than CAS, although the CAS diet also modulated the intestinal microbiota to a composition compatible with improved bowel movement frequency and lipid metabolism.

## 1. Introduction

The intestinal gut microbiota shaped by animal or vegetable proteins have been shown to differently influence puberty timing [1], thus encouraging soy protein intake in adolescents. This is just one example of the growth of interest in soy, and other alternative sources of vegetable proteins such as legumes, for human nutrition in recent decades. Indeed, the health and environmental implications of excess animal proteins in Western diets encourage the consumption of other protein sources, which include legumes as the most valuable alternative [2]. Regular legume consumption has been reported to contribute to a reduction in the risk of cardiovascular disease, through the modulation of blood pressure, plasma lipid levels and inflammation, and regulation of glucose metabolism and body weight, with a consequent decrease in the risk of developing type II diabetes [3]. In this context, legume seeds of the genus *Lupinus* are a potentially relevant protein sources for human nutrition. White lupin (*Lupinus albus*) is of particular interest due to its high protein, high fibre, and low fat content; presence of potentially beneficial bioactive compounds; health benefits of its consumption including those in energy metabolism, cardiovascular disease, bowel function, and anticonvulsant actions; and its agronomic importance as a soil N fixing crop [4,5].

Legume proteins, including those of lupin, are generally considered inferior in nutritional quality compared with animal proteins such as casein (CAS) or lactalbumin (LA), mainly because of their lower content of essential amino acids (EAA), particularly sulphur amino acids (AA) [6,7]. Additionally, even after EAA supplementation, the nutritional quality of *L. angustifolius* proteins has been reported to be lower than LA, possibly due to effects of lupin protein on nitrogen metabolism. In fact, under normal feeding conditions, absorption of AA from lupin protein isolates (LPI) occurs at slower rates than that of animal proteins, and this might explain the lower nutritional utilization of legume storage proteins compared with milk proteins [8]. All these considerations notwithstanding, lupin protein have been reported to display an adequate nutritional value when used in properly supplemented diets [9].

In addition to cardiovascular disease, legume consumption as part of the Mediterranean diet has been associated with a lower risk of other pathological conditions including colorectal cancer, and recent studies have associated intestinal microbiota (IM) composition shaped by legume protein consumption to the prevention of intestinal inflammation, a process commonly associated with cancer [10]. However, information on the effects of specific chemical fractions or components in the highly complex gastrointestinal environment is still lacking. Studies in animal models are needed to investigate the effect of specific dietary components—including proteins—thus allowing the development of new molecules or dietary manipulations.

Among the various food nutrients, attention on dietary proteins which account for a substantial part of the human diet (up to 30%, 70–100 g of protein/day) is increasing, since in the distal colon, where protein fermentation mostly occurs, toxic substances detrimental for health and implicated in bowel disease and colon cancer (hydrogen sulphide, a number of phenolic and indolic compounds, and ammonia) are produced [11]. In the case of daily protein intake being 2–5 times greater than the dietary recommendations, shifts toward increased proteolytic fermentation can generate metabolic products capable of altering the relative abundance of microbial species in the gut, and increase the intestinal inflammatory response, tissue permeability, and colitis severity. These metabolic products have also been linked to the development of colorectal cancer and metabolic diseases, including obesity, diabetes, and non-alcoholic fatty liver disease [12].

Increasing protein intake in athletes on weight loss diets is among the recommended strategies to counteract the negative net muscle protein balance. Whey, and particularly its main protein component alpha-lactalbumin, has been shown to play a role in promoting high-quality weight loss during caloric restriction [13]. Investigations into the dietary components which may affect the microbiota, and microbiota groups potentially able to mediate the effects of individual dietary components on the human health are future challenges in this area of research. It is known that many bacterial protein degradation substances produced after protein degradation are toxic or detrimental to gut health [14,15]. However, the dietary carbohydrate component has been repeatedly shown to strongly influence the intestinal microbial composition/functionality, which is mainly due to the wide array of enzymes encoded in our intestinal bacteria able to degrade and ferment a variety of polysaccharides and glycans, mostly of dietary origin, that enter the large intestine [16,17]. It is probably for this reason that the colonic fermentation of carbohydrates has received much more attention than proteins [16].

As indicated above, protein isolates from *L. albus* have potential as novel human food ingredient [9], and a *L. angustifolius* protein isolate has been recently found to affect the intestinal microbiota composition in animal models [18]. The aim of the present study was to evaluate the effect of an *L. albus* protein isolate (LPI)-based diet on the intestinal microbiota composition, and to compare such effect with that of diets based on casein (CAS) or lactalbumin (LA) as protein constituents. Accordingly, energy and protein equalised semi-synthetic diets for rats, differing only in their protein component (i.e., LA, CAS, LPI), were produced to study their effect on faecal microbiota composition in the absence of other dietary components.

## 2. Materials and Methods

### 2.1. Diets

Lupin protein isolate Type E (LPI) was manufactured by Fraunhofer Gesellschaft, Fraunhofer-Institute (IVV) (Munich, Germany) as in [19] by using white lupin (*L. albus*) seeds (low-alkaloid, de-oiled). In summary, LPI was manufactured using an extraction/precipitation process followed by drying of the resultant LPI which consists predominantly of globulins (7S and 11S fractions).

The diets, manufactured at Laboratory Piccioni (Milano, Italy) and previously described [9], contained corn oil as a fat source, and corn starch, potato starch and glucose as carbohydrate sources. Diets were supplemented with vitamins and minerals to meet requirements [20]. LA, CAS and LPI were the sole protein source of each diet. The EAA composition of LA, CAS (Sigma Chemical Co., Ltd., Alcobendas, Madrid, Spain) and LPI, determined by HPLC, is given in Table 1 [21]. Diets were manufactured at Laboratory Piccioni (Milano, Italy) based on each of LA, CA and LPI as the sole protein source. The three diets were formulated to contain the same amounts of protein (100 g/kg) and equal digestible energy (15.4–15.5 kJ/g). Appropriate amounts of synthetic EAA were added to the LPI and CAS diets to bring the levels to those of the LA diet (Table 2).

### 2.2. Animals and Treatments

As previously described [9], male weaned Sprague-Dawley rats were housed in groups of 3–4 animals per cage and fed the LA-containing diet ad libitum for 14 days as acclimatization period. Animals were then divided into three groups of ten animals each and fed ad libitum, for 28 days, LA, CAS or LPI diet. On day 28th of the dietary treatment, faeces were collected, immediately frozen in liquid nitrogen, and stored at −80 °C prior to freeze-drying and DNA extraction [18] Animals were then sacrificed by using humanitarian methods (see ARRIVE report).

Procedures involving animals and their care were conducted in accordance with institutional guidelines that are in compliance with national and international laws and policies. The experimental protocol was approved by the Italian Ministry of Health (Protocol No. 2006/3). The ARRIVE report was provided.

### 2.3. RT-qPCR Microbiota Composition Analysis

Freeze-dried faecal samples were finely ground and stored at −80 °C until use, and the total DNA was isolated from 40 g samples by using the FavorPrep Stool DNA Isolation Mini Kit (Favorgen-Europe, Vienna, Austria), and following the manufacturer’s instructions. Treatment of eluted DNA, assessment of DNA concentration, and log_10_ number of copies determination by using quantitative polymerase chain reaction (q-PCR) (iQ5 Cycler, Bio-Rad Laboratories, Alcobendas, Spain) were as in [18]. Samples for q-PCR analysis were run in duplicate.

### 2.4. High-Throughput Analysis of Microbial Community

Illumina technology (MiSeq, Emeryville, CA, USA) was used to determine bacterial diversity. Ten rats per group (*n* = 30) were used to isolate total DNA from freeze-dried faecal samples as described above. Amplification of the V3–V4 region of the 16S rRNA gene was used for libraries preparation by using primers 5′ CCTACGGGNGGCWGCAG 3′ and 5′ GACTACHVGGGTATCTAATCC 3′ for amplification. PCR were run in duplicate, and conditions were as in [18].

### 2.5. Analyses of Predicted Microbial Functions

Prediction of functional gene compositions of bacterial communities was assessed by the PICRUSt (phylogenetic investigation of communities by reconstruction of unobserved states) method [23] and QIIME2 (version 2021.11) with the GreenGenes V13.8 database were used to generate BIOM-formatted files for taxonomic classification [24]. The KEGG orthologs database [25] was used for functional prediction and summarized at the pathway hierarchy level 3.

### 2.6. Statistical Analysis

Results from high throughput sequencing analyses were performed by using QIIME2 (version 2021.11). Quality filtering, Deblur, and alignment and taxonomic assignation were performed against the Greengenes database as in [18]. Sub-OTUs obtained by MiSeq Illumina analysis (Emeryville, CA, USA) were grouped by bacterial species (obtained from the bar plots produced by QIIME2). Multivariate statistical techniques and Bray–Curtis measures of similarity were used to explore the similarities in rat faecal microbiota and identify species which account for differences observed in these bacterial communities. Differences in gut microbial groups between treatments were tested by Analysis of Similarity (ANOSIM), and the relationships between bacterial groups by Principal Component Analysis (PCA), after Analysis of Similarity Percentages (SIMPER), which was carried out to determine the overall average similarity in faecal microbial community compositions. Differences among the different dietary groups were studied by Discriminant Analysis (DA). The Linear Discriminant Analysis Effect Size (LEfSe) method was used to test differences in the abundance of families, genera, and functional categories [26] with an alpha value of 0.05 for the Kruskal–Wallis test among classes, and the threshold for the log_10_LDA score was set at 2.0.

## 3. Results

At the end of the dietary treatments, the body weight of the rats fed LPI was 16.9% higher (*p* < 0.05) than that of rats fed LA. No other significant differences were observed in growth parameters, including food intake [9]

### 3.1. RT-qPCR Microbiota Composition

**Figure 1:**
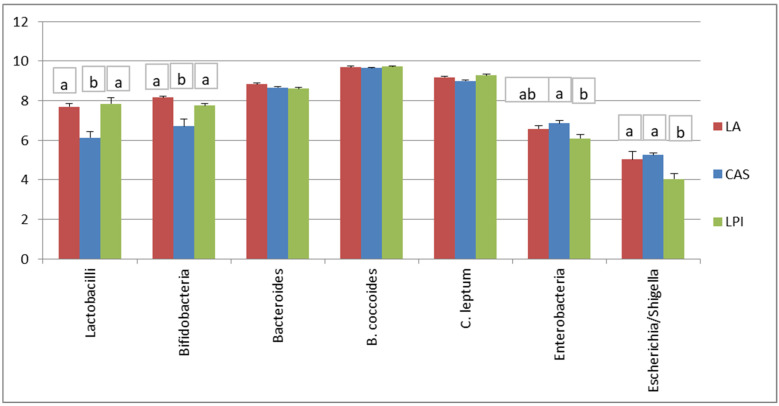
Bacterial counts (log_10_ copies of the 16 S-rRNA gene mg^−1^ dry content) in the faecal content of rats fed diets containing milk-derived (LA, CAS) or lupin protein isolate (LPI) as the only protein component. Data are expressed as mean ± SD in bars (*n* = 10). Different letters indicate significant (*p* < 0.05) differences.

Figure 1 shows faecal bacteria log_10_ copy numbers of rats fed the different experimental diets. The CAS diet induced lower Total bacteria, *Bifidobacteria* and *Lactobacilli* compared to LA or LPI, while the LPI diet gave place to lower *Enterobacteria* than CAS, and lower *Escherichia*/*Shigella* than LA or CAS.

### 3.2. Results on High-Throughput Analysis 

Reads (1,184,882) from the 30 faecal samples were processed through Illumina MiSeq technology. High quality sequences (629,751) obtained after Deblur belonging to 585,084 OTUs and 187 bacterial species were retained for subsequent analyses. Bacterial groups with higher contribution to dissimilarity were selected by a similarity percentages breakdown (SIMPER analysis) was used. As a consequence, 26 species (*Allobaculum* spp., spp. from *Clostridiales*, spp. from *Lachnospiraceae*, spp. from *Bacteroidales*, spp. from *Ruminococcaceae*, *Ruminococcus* spp., *R. bromii*, spp. from *Rickenellaceae*, *Parabacteroides* spp., *Collinsella aerofaciens*, spp. from *Alphaproteobacetria*, *Bifidobacterium* spp., *B. animalis*, spp. from *Erysipelotrichaceae*, *Bacteroides* spp., spp. from *Peptostreptococcaceae*, *Lactobacillus* spp., *L. reuteri*, *Akkermansia muciniphila*, spp. from *Clostridiaceae*, *Blautia* spp., *B. producta*, *Turicibacter* spp., *Sutterella* spp., *Eubacterium dolichum* and spp. from *Enterobacteriaceae*) belonging to 22 genera were responsible for >90% of the dissimilarity.

**Figure 2:**
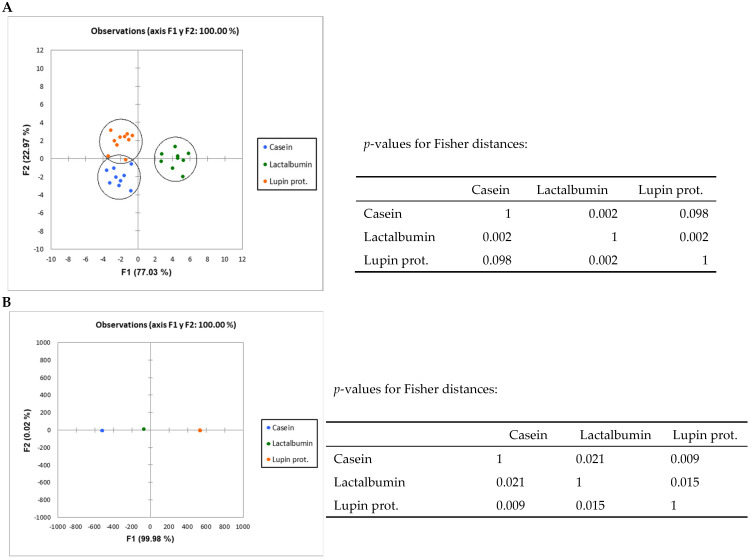
Discriminant Analysis of bacterial groups analysed by Illumina sequencing; LA, lactalbumin; CAS, casein; LPI, lupin protein isolate. (**A**) family level; (**B**) species level.

The three diets led to significant (*p* < 0.01) differences in the faecal microbiota composition in all comparisons as shown by ANOSIM analysis (Table 3) of the high throughput results. In addition, discriminant analysis (Figure 2) evidenced that the diets were grouped differently at both the family and genus levels.

As shown in Table 4, *Bacteroidetes*, *Firmicutes*, *Proteobacteria*, *Actinobacteria*, *Verrucomicrobia* and *Tenericutes* were the most abundant phyla in all treatments. Interestingly, the *Firmicutes*/*Bacteroidetes* (F/B) ratio was higher for the LPI diet compared to LA and CAS. At the genus level (Table 4), the LA group had higher *Collinsella*, and lower genera from *Peptostreptococcaceae* and *Clostridiaceae*; the CAS group had higher Parabacteroides and genera from *Enterobacteriaceae*, and lower *Lactobacillus*; the LPI group had higher genera from *Clostridiaceae* and *Erysipelotrichaceae*, and lower genera from *Alphaproteobacteria*, *Blautia*, *Suterella* and genera from *Enterobacteriaceae*. At the species level (Table 4), the LA group had higher *Collinsella aerofaciens*, and lower species from *Peptostreptococcaceae* and from *Clostridiaceae*; the CAS group had higher *Parabacteroides* spp. and genera from *Enterobacteriaceae*, and lower *Lactobacillus* spp. and *L. reuteri*; the LPI group had higher species from *Clostridiaceae* and *Erysipelotrichaceae*, and lower species from *Alphaproteobacteria*, *Blautia* spp. and *B. producta*, *Suterella* spp. and species from *Enterobacteriaceae*.

**Figure 3:**
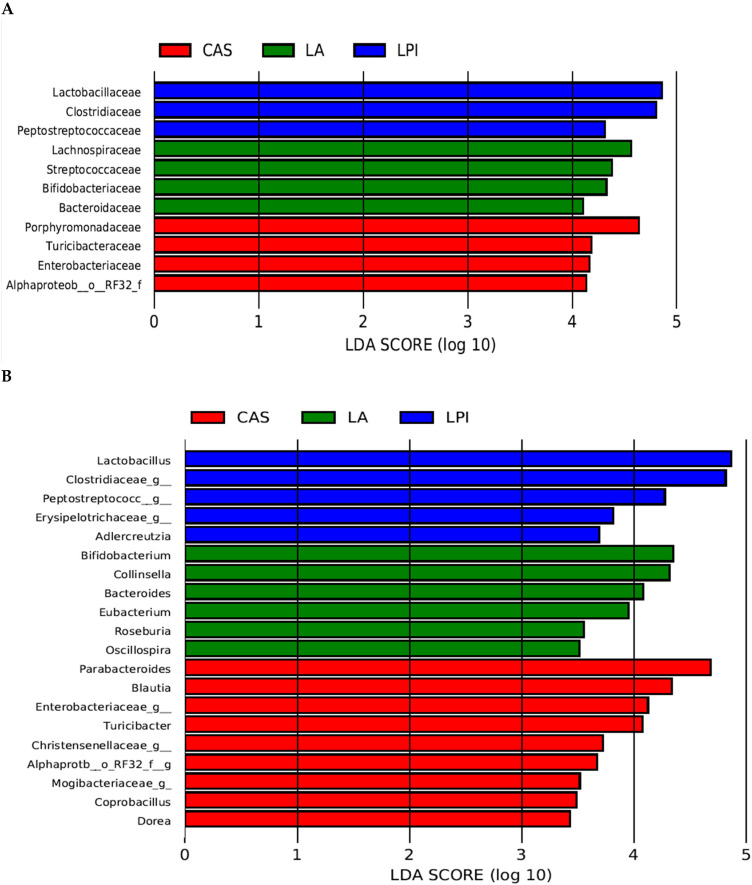

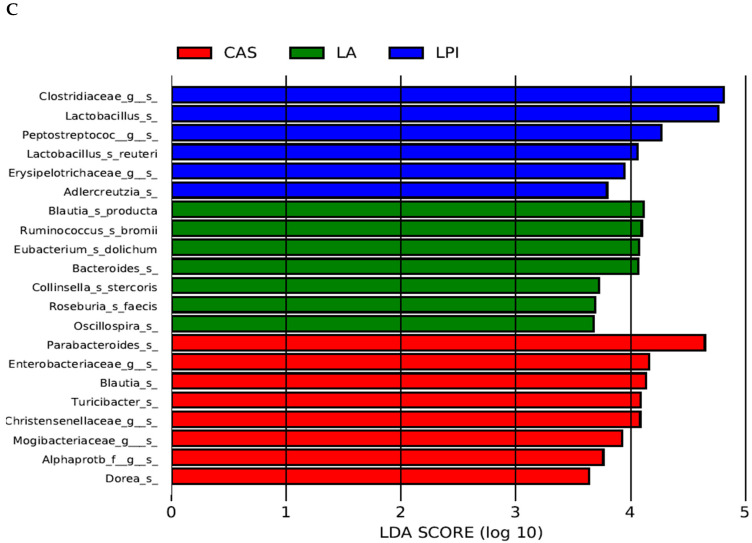
Linear discriminant analysis coupled with effect size (LEfSe) of groups after SIMPER analysis, using the default parameters (LDA score = 2). (**A**) family level; (**B**) genus level; (**C**) species level.

The LEfSe algorithm has been proven as effective in detecting differentially abundant features in the microbiome by allowing the characterization of microbial taxa specific to an experimental or environmental condition, the detection of pathways and biological mechanisms over- or under-represented in different communities, and the identification of metagenomic biomarkers in mammalian microbiomes [26]. As shown in Figure 3, the organisms most likely to explain differences in the LA group were *Bifidobacterium* spp., *Collinsella* spp. (in particular *C. stercoris*), *Bacteroides* spp., *Eubacterium* spp. (in particular *E. dolichum*), *Roseburia* spp. (in particular *R. faecis*), and *Oscillospira* spp. In the case of the CAS group, the organisms were *Parabacteroides* spp., *Blautia* spp., *Enterobacteriaceae* spp., *Turicibacter* spp., *Christenellaceae* spp., species from *Alphaproteobacteria* and *Mogibacteriaceae*, *Coprobacillus* spp. and *Dorea* spp. In the case of the LPI group, the organisms were *Lactobacillus* spp. (*Lactobacillus* spp. and *L. reuteri*), species from *Clostridiaceae*, species from *Peptostrptococcaceae*, species from *Erysipelotrichaceae*, and *Adlercreutzia* spp.

**Figure 4:**
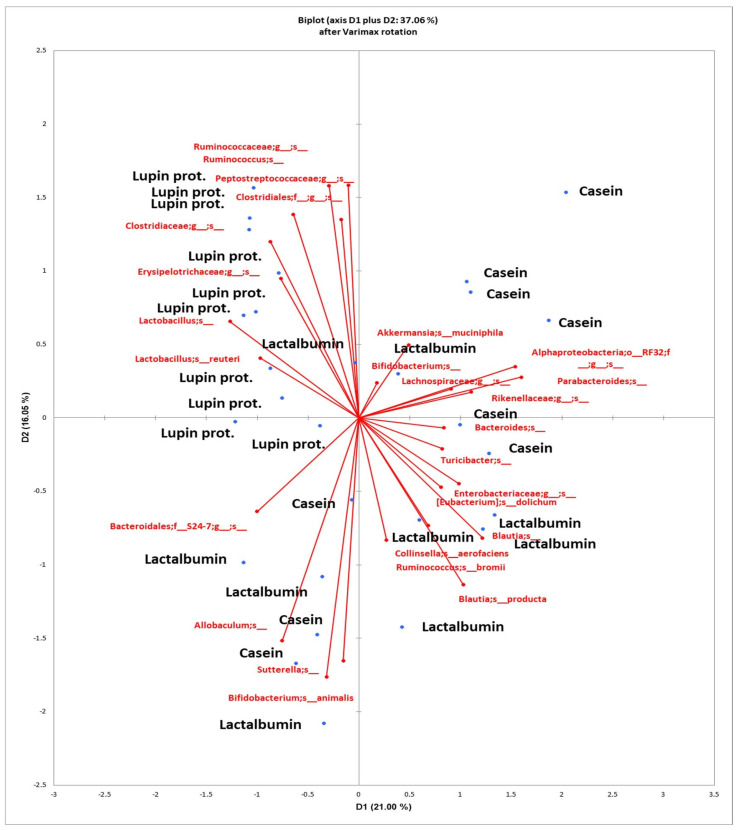
Principal Components Analysis (PCA) of groups at the species level (after Varimax rotation and SIMPER analysis) of the sequencing analysis results of the bacterial community of faecal samples of rats fed diets based in milk (LA, CAS) or lupin proteins isolate (LPI) as the only protein source. Treatments in black, bacterial groups in red.

The PCA of the Illumina results (Figure 4) at the species level also separated LPI from LA and CAS, which were closer to each other. A number of species (*Ruminococcaceae* and *Ruminococcus* spp., *Peptostreptococcaceae*, *Clostridiaceae*, *Erysipelotrichaceae*, *Lactobacillus* spp. and *L. reuteri*) clustered with the LPI diet.

ANCOM-BC (Analysis of Compositions of Microbiomes with Bias Correction) tests hypothesis regarding differential absolute abundance of individual taxon and provides valid confidence [27]. By using this procedure, we identified key bacterial species able to discriminate among the different diets. As shown in Figure S1, at the genus level *Lactobacillus reuteri* (W = 180, LPI diet), and *Lactococcus* spp. (W = 173, LA diet) showed a significant difference (*p* < 0.05) in abundance in the rats’ faecal microbiota.

By using PICRUSt, the predicted functions of the intestinal microbiota were identified. The functionality of the intestinal microbiome in rats fed the CAS differed from LA but not from LPI, while LA differed from both CAS and LPI, as shown by discriminant analysis (Figure S2) of the PICRUSt analysis. However, no significant differences were found in individual predicted functions among groups (Figure S3).

## 4. Discussion

In comparison with other *Lupinus* spp. used as crops, seeds from *L. albus* have been found to be the highest in oil content, and to have a more beneficial AA composition from a nutritional point of view, as well as fewer alkaloids than blue (*L. angustifolius*) or yellow (*L. luteus*) lupin species. Regarding the protein fraction, all of the species are deficient in methionine, and show lysine, tryptophan and valine levels below the standards of nutrition. However, white lupin is characterised by a higher EAA index and chemical score of restrictive AA, as well as the highest protein efficiency ratio (PER). White lupin is therefore considered the most suitable for human and animal nutrition, as well as for protein supplements production [7]. Furthermore, the potential usefulness of these protein concentrates requires to be tested preferentially in in vivo models. In this line, we have conducted previous work to study the nutritional value [9], together with some physiological effects in rats fed diets based on isolated lupin proteins as the only protein source [8,9]. White lupin proteins have shown beneficial physiological effects in a wide range of unfavourable conditions such as diabetes, hypertension, obesity, and cardiovascular diseases, and they may ease the glycemia control in diabetics or pre-diabetics [28,29,30].

The nutritional value, gastrointestinal processing and the utilization of these or other proteins—as with any other dietary component—is known to be mediated by the activity of the microbiota residing within the intestinal tract. On the other hand, the bacterial species with the greatest capacity for proteolytic fermentation cannot be solely identified in a non-competitive in vitro environment, but would instead require a model as close as possible to the highly competitive intestinal environment [12]. In line with this, we have recently reported that different proteins, including a lupin protein isolate from *L. angustifolius*, do modulate the intestinal microbiota composition in rats [18]. As vegetable protein concentrates are increasingly been considered in a variety of dietary formulations (for example muscle hypertrophy among strength athletes such as bodybuilders and powerlifters; in recovery from intense exercise; in energy-restricted weight loss diets, etc.), and given the broad implications of the shifts in intestinal microbiota composition, it becomes increasingly relevant concentrates—to explore the effects of including this particular type of protein concentrates on the intestinal microbiota balance and/or metabolism. However, published work can scarcely be found that is specifically aimed to systematically compare the effects on the intestinal microbiota composition and function induced in vivo by chemically defined proteins, without the interference of other dietary components. The proteins here utilized for comparison with LPI were those mostly used as control proteins in nutritional studies with rats (LA and CAS). The three diets were formulated to contain the same amounts of protein (100 g/kg) and equal digestible energy (15.4–15.5 kJ/g). Significant differences in composition and functionality were revealed by qPCR and Illumina sequencing analysis after feeding diets based in different food proteins. Thus, as shown in Table 3 and Figure 2, ANOSIM and Discriminant Analysis elicited very significant differences among the different dietary groups at both family and genus levels.

To summarise the results found in the current investigation, as shown in Table 4, the three proteins tested here gave place to significant differences in faecal microbiota composition. Thus, the LPI group had higher values for species from *Clostridiaceae* and *Erysipelotrichaceae*, and lower values for species from *Alphaproteobacteria*, *Blautia* spp. and *B. producta*, *Suterella* spp. and species from *Enterobacteriaceae*; the LA group had higher *Collinsella aerofaciens*, and lower species from *Peptostreptococcaceae* and *Clostridiaceae*; and the CAS group had higher *Parabacteroides* spp. and genera from *Enterobacteriaceae*, and lower *Lactobacillus* spp. and *L. reuteri*. These results are in keeping with those from the qPCR analysis (Figure 1) where the CAS diet feeding resulted in lower *Bifidobacteria* and *Lactobacilli* compared to the other diets, and the LPI diet gave way to lower *Enterobacteria* than CAS, and lower *Escherichia*/*Shigella* than LA and CAS. On the other hand, LEfSe analysis (Figure 3) revealed that the organisms most likely to explain differences in the LA group were *Bifidobacterium* spp., *Collinsella* spp. (in particular *C. stercoris*), *Bacteroides* spp., *Eubacterium* spp. (in particular *E. dolichum*), *Roseburia* spp. (in particular *R. faecis*), and *Oscillospira* spp. In the case of the CAS group, the organisms were *Parabacteroides* spp., *Blautia* spp., *Enterobacteriaceae* spp., *Turicibacter* spp., *Christenellaceae* spp., species from *Alphaproteobacteria* and *Mogibacteriaceae*, *Coprobacillus* spp. and *Dorea* spp. In the case of the LPI group, the organisms were *Lactobacillus* spp. (*Lactobacillus* spp. and *L. reuteri*), species from *Clostridiaceae*, species from *Peptostreptococcaceae*, species from *Erysipelotrichaceae*, and *Adlercreutzia* spp., which is in keeping with the PCA results (Figure 4).

Lower qPCR *Enterobacteria* and *Escherichia/Shigella* values than LA and CAS were found for the LPI diet (Figure 1). This was in line with results from sequencing (Table 4) and LEfSe analysis (Figure 3), and also with previous work by our group in rats or pigs fed legume-based diets, where lower *Enterobacteria* and *Escherichia*/*Shigella* were found [31,32]. Rist et al. [33] reported that piglets fed diets based in highly digestible casein showed higher *Enterobacteriaceae* counts than piglets fed soybean meal-based diets. This is in line with previous work by our group [33] where the number of copies for Lactobacilli and Bacteroides in pigs fed on a casein-based diet was lower than soybean, but was higher than soybean for *Bifidobacteria*, *Enterobacteria* and the *Escherichia*/*Shigella* group. The potential pathogenicity of Enterobacteria is widely recognised, and several genera belonging to the *Enterobacteriaceae* family are considered fatal pathogens because of their resistance to antibiotics and their implication in a variety of diseases [34]. However, since in most previous reports protein isolates were not used in diet formulation, the diets used differed also in the composition of their carbohydrate fraction, making it difficult to ascribe the effects found mainly or solely to the protein component of the diets. The main driver of intestinal microbiota composition is usually ascribed to the dietary proportion of proteins to carbohydrates contents [15]. Thus, for example, mice fed a high-protein and low-carbohydrate diet resulted in increased proportions of *Bacteroides* spp. and *Parabacteroides* spp., while the families *Lachnospiraceae* and *Ruminococcaceae* were decreased, which may result in a deleterious gut environment [35]. In addition, other components such as polyphenols are known to have a substantial effect [15]. For this reason, it is important to establish which effects mainly result from changes in the protein fraction of the diet. In connection with this, it is noteworthy that the results reported here are very much in line with those recently published [18], where rats fed an isolated lupin protein had lower *Escherichia*/*Shigella* amounts compared with the CAS diet. This is probably even more relevant taking into account that proteins from a different lupin species (*L. angustifolius*) were used in that previous report. Interestingly, a lower weight of cecum and total large intestine relative to body weight was observed in rats fed the white lupin isolate compared with casein- and lactalbumin-fed animals [9]. This is likely to be linked to an anti-inflammatory bacterial population, which is relevant to colon cancer risk reduction. In addition, that may be associated with reduced colon cancer risk in humans [10] since legumes are included as an important part of the “prudent” diet. So, the effect of lupin or other legume proteins on reduction in large intestinal weight and the relationship between intestinal weight changes and intestinal cell proliferation/carcinogenesis is worthy of further investigation.

The CAS diet resulted in lower qPCR lactobacilli values than LA and LPI (Figure 1). This was in line with results from sequencing (Table 4), where higher *Lactobacillus* spp. and *L. reuteri* were found for the LPI diet, and with LEfSe (Figure 3) and ANCOM analysis (Figure S1), where *Lactobacillus* spp. and *L. reuteri* were identified as the differentially abundant groups. The probiotic effect of different *Lactobacillus* spp. strains in diverse models of murine or human colitis is widely recognised, and their mode of action includes inhibition of the growth of certain pathogens, such as colitogenic microbes (*Enterobacteria*, *E. coli*) and modulation of mucosal and/or systemic immune response or metabolic functions [36]. The mechanism of action of probiotics, most of which are Lactobacilli and Bifidobacteria, has been linked to microbiota modulation, alteration of gut barrier function, visceral hypersensitivity, gastrointestinal dysmotility, intestinal immunological modulation, and microbiota–gut–brain axis communication and comorbidities [37]. In particular, the interest of *L. reuteri* as a probiotic has been highlighted on intestinal disorders in humans, and together with a tryptophan-rich diet, it was able to reprogram intraepithelial T cells into immuno-regulatory T cells and play a relevant role in the immune system maturation [38].

Most of the organisms pointed out by LEfSe in the LA group in the current work [*Blautia* spp. (in particular *B. producta*), *Ruminococcus* spp. (in particular *R. bromii*), *Eubacterium* spp. (in particular *E. dolichum*), *Bacteroides* spp. (in particular *C. aerofaciens*), *Roseburia* spp. (in particular *R. faecis*), and *Collinsella stercoris*] coincide with those found previously [18] and have been identified as butyrate producers [39,40]. Butyrate, together with acetate and propionate, the three major SCFAs, can be produced from AA in the distal colon, but are also the main end products from carbohydrates fermentation in the proximal colon, being butyrate, the major energy source for epithelial cells, since 70–90% of it is metabolized in the colonocytes. In addition, SCFAs exert anti-inflammatory effects on the intestinal epithelial cells, and regulate metabolism through binding to G-protein coupled receptors [41]. Also, increased SCFAs production is known to lower the colon luminal pH, which substantially prevents the overgrowth of pH sensitive pathogenic bacteria such as *Escherichia* and some *Clostridia* [11]. On the contrary, some species from other groups (*Enterobacteriaceae*) pointed out for CAS (*Parabacteroides* spp., *Enterobacteriaceae* spp., *Turicibacter* spp., *Christenellaceae* spp., species from *Alphaproteobacteria* and *Mogibacteriaceae*, *Coprobacillus* spp. and *Dorea* spp.) have been reported as pathogenic or potentially pathogenic (see above). However, Oki et al. [42] reported that the abundances of *Christensenellaceae*, *Mogibacteriaceae*, and *Rikenellaceae* were negatively correlated with bowel movement frequency, and that the abundances of these bacterial families were significantly higher in lean subjects (BMI < 25). Interestingly, these three groups were among those specifically relevant in the CAS fed rats (Figure 3C and Table 4). Moreover, the abundances of these families were associated with a lower level of triglyceride (*Christensenellaceae* and *Rikenellaceae*) and higher level of HDL cholesterol (*Mogibacteriaceae*) both of which correlate with lower BMI [43]. Both bowel movement frequency and especially lipid metabolism are of great relevance in human health, and therefore these results may deserve closer attention. Thus, according to the current research, LA appears to induce a generally healthier microbiota composition than CAS (lower *Enterobacteriaceae* and higher Lactobacilli and butyrate producers) in the absence of other dietary components, and would therefore seem preferable in high protein diets, although CAS gave place to a cohort of metabolically active bacterial groups with potentially positive health implications. In addition, it is worth mentioning that the results from the previous work [19] were confirmed here even though seeds from a different lupin species (*L. angistifolius*), and different experimental design (different protein extraction procedure, different animal procedures) were used.

Finally, it should be kept in mind that we have used a rodent model here, which is not without limitations when extrapolated to human physiology. In addition, we used diets specifically formulated for rodents where only a limited number of ingredients were incorporated into the mix, and which is far from the usual situation of human eating behaviour. Therefore, these results should be taken with caution before well-designed short and/or prospective studies are actually carried out in humans.

## 5. Conclusions

In the present study, the effect of a *L. albus* protein-based diet on the intestinal microbiota composition was investigated for the first time, and it was compared to that of diets based on lactalbumin and casein as protein sources. Both qPCR and Illumina sequencing analysis showed significant differences in microbiota composition and functionality in rats fed diets differing only in their constituent proteins, in line with results recently published. Despite differences in the experimental procedures used here, proteins from *L. albus* were found to induce a beneficial intestinal microbiota profile compared to that of milk protein-based diets, as found with the lupin isolate previously investigated (*L. angustifolius*). In addition, in the absence of other dietary components, lactalbumin appeared to induce a generally healthier microbiota composition than casein according to the current research. This would make lactalbumin preferable to casein in high protein diets, which is also in keeping with the results previously reported. On the other side, casein also modulated the intestinal microbiota to a composition compatible with improved bowel movement frequency and lipid metabolism. Finally, it was confirmed here that the constituent protein fraction of the meal is likely to be at least in part responsible for the beneficial modulation of intestinal microbiota generally found with legume-based diets.

## Figures and Tables

**Table 1:** EAA composition of LA, CAS and LPI (g/kg).

Amino Acids	LA ^1^	CAS	LPI
Histidine	14.1	27.5	17.9
Isoleucine	40.2	40.9	39.1
Leucine	65.3	74.9	60.0
Lysine	80.1	97.9	47.5
Methionine + Cysteine	55.5	39.2	15.3
Phenylalanine + Tyrosine	46.3	95.8	83.4
Threonine	50.0	39.6	30.0
Tryptophan ^2^	16.7	10.0	5.0
Valine	37.4	53.3	30.2

^1^ LA, lactalbumin; CAS, casein; LPI; lupin proteins isolate. ^2^ Literature values [20,22].

**Table 2:** Protein amount and EAA supplementation of LA, CAS and LPI diets (g/kg).

Diet	LA ^1^	CAS	LPI
Lactalbumin	138	-	-
Casein	- ^2^	118	-
Lupin proteins isolate	-	-	128
EAA			
Lysine	-	-	5.0
Methionine	-	1.9	4.6
Threonine	-	-	0.3
Tryptophan	-	-	1.0
Valine	-	-	1.1

^1^ LA, lactalbumin; CAS, casein; LPI; lupin proteins isolate. ^2^ - Not added

**Table 3:** ANOSIM (distance measure: Bray–Curtis, Bonferroni corrected *p* values) on proportions of sequencing results at different taxonomic levels of samples from the faecal bacterial community of rats fed diets containing milk-derived (LA, CAS) or lupin proteins isolate (LPI) as the only protein component (*n* = 10).

Taxonomy	Diet ^1^		
	LA	CAS	LPI
**Family**			
LA	0	0.023	0.001
CAS		0	0.001
LPI			0
**Genus**			
LA	0	0.021	<0.001
CAS		0	<0.001
LPI			0
**Species**			
LA	0	0.021	<0.001
CAS		0	<0.001
LPI			0

^1^ LA, lactalbumin. CAS, casein; LPI, lupin protein isolate.

**Table 4:** Proportions of Illumina sequencing reads at different taxonomic levels of the faecal bacterial community of rats fed diets containing milk-derived (LA, CAS) or lupin proteins isolate (LPI) as the only protein source. “*f__*”, “*g__*” and “*s__*” indicate unknown Family, Genus and Species, respectively.

Taxonomy	Diet ^1^			
	LA ^2^	CAS	LPI	*p* Values
**Phylum**				
Bacteroidetes	4900	3830	2755	0.231
Firmicutes	14,091	9824	17,882	0.514
Proteobacteria	1025 ab	1201 a	410 b	0.085
Actinobacteria	2406 a	1229 ab	1007 b	0.085
Verrucomicrobia	1081	850	892	0.838
Tenericutes	29	49	136	0.198
F/B	3 a	3 a	5 b	0.006
**Genus**				
*Allobaculum*	491	502	458	0.947
*Clostridiales;f__;g__*	124 ab	103 b	187 a	0.072
*Lachnospiraceae;g__*	178	142	132	0.175
*Ruminococcaceae;g__*	138	163	186	0.273
*Bacteroidales;f__S24-7;g__*	331	146	258	0.119
*Ruminococcus*	165	150	195	0.533
*Rikenellaceae;g__*	106 ab	154 a	65 b	0.049
*Parabacteroides*	321 b	615 a	275 b	0.005
*Collinsella*	239 a	4 b	1 b	<0.0001
*Alphaproteobacteria;o__RF32;f__;g__*	26 a	36 a	2 b	<0.0001
*Bifidobacterium*	315 a	245 ab	182 b	0.109
*Erysipelotrichaceae;g__*	13 b	5 b	42 a	<0.0001
*Bacteroides*	137 a	128 a	23 b	0.004
*Peptostreptococcaceae;g__*	73 b	192 a	205 a	0.002
*Lactobacillus*	447 a	107 b	652 a	0.001
*Akkermansia*	214	270	258	0.796
*Clostridiaceae;g__*	122 c	372 b	576 a	<0.0001
*Blautia*	196 a	198 a	37 b	0.002
*Turicibacter*	122 a	136 a	47 a	0.097
*Sutterella*	86 ab	94 a	44 b	0.044
*[Eubacterium]*	78 a	39 ab	7 b	0.054
*Enterobacteriaceae;g__*	62 b	122 a	10 c	<0.0001
**Species**				
*Allobaculum;s__*	491	502	458	0.947
*Clostridiales;f__;g__;s__*	124 ab	103 b	187 a	0.072
*Lachnospiraceae;g__;s__*	178	142	132	0.175
*Bacteroidales;f__S24-7;g__;s__*	331	146	258	0.119
*Ruminococcaceae;g__;s__*	138	163	186	0.273
*Ruminococcus;s__*	54 b	133 ab	138 a	0.072
*Ruminococcus;s__bromii*	117	59	38	0.141
*Rikenellaceae;g__;s__*	106 ab	154 a	65 b	0.049
*Parabacteroides;s__*	350 b	615 a	275 b	0.006
*Collinsella;s__aerofaciens*	114 a	0.2 b	0.3 b	0.030
*Alphaproteobacteria;o__RF32;f__;g__;s__*	29 a	32 a	2 b	0.001
*Bifidobacterium;s__*	180	99	151	0.485
*Bifidobacterium;s__animalis*	196 a	85 ab	52 b	0.064
*Erysipelotrichaceae;g__;s__*	13 b	5 b	49 a	<0.0001
*Bacteroides;s__*	137 a	128 a	23 b	0.004
*Peptostreptococcaceae;g__;s__*	73 b	192 a	205 a	0.002
*Lactobacillus;s__*	381 b	106 c	627 a	<0.0001
*Lactobacillus;s__reuteri*	66 a	1 b	101 a	0.001
*Akkermansia;s__muciniphila*	192	270	235	0.655
*Clostridiaceae;g__;s__*	122 c	372 b	576 a	<0.0001
*Blautia;s__producta*	110 a	86 a	32 b	0.015
*Blautia;s__*	111 a	112 a	5.200 b	0.001
*Turicibacter;s__*	109 ab	152 a	52 b	0.100
*Sutterella;s__*	86 ab	94 a	49 b	0.086
*[Eubacterium];s__dolichum*	78 a	39 ab	7 b	0.054
*Enterobacteriaceae;g__;s__*	48 b	122 a	16 b	<0.0001

Only groups selected after SIMPER analysis and with a contribution higher than 1% were included. ^1^ LA, lactalbumin; CAS, casein; LPI, lupin protein isolate. ^2^ Values are means of 10 animals per group. ^a,b,c^ Means not sharing superscript letters differ significantly (*p* < 0.05) or tend to be different (*p* < 0.1).

## Data Availability

Data may be provided by authors on request.

## Supplementary Materials

The following supporting information can be downloaded at: www.mdpi.com/article/10.3390/nu17030551/s1, Figure S1: ANCOM analysis of the samples from the faeces of rats fed LA, CAS or LPI diets. The clr (centered log ratio) transformed OTU table at the species level that was modified to adjust 0 values to 1 was used. The W value represents the number of times of the null hypothesis (the average abundance of a given species in a group is equal to that in the other group) was rejected for a given species. Only species with reject null-hypothesis >95% are labelled; Figure S2: Discriminant Analysis of PICRUSt predicted functions after SIMPER (50% dissimilarity) analysis of the intestinal microbiota bacterial groups analysed by Illumina sequencing; LA, lactalbumin; CAS, casein; LPI, lupin proteins isolate; Figure S3: ANOVA of PICRUSt functional analysis after SIMPER (50% dissimilarity) analysis, using the default parameters (LDA score = 2).

## Abbreviations

AA, amino acids; EAA, essential AA; CAS, casein; LA, lactalbumin; LPI, lupin protein isolate; SCFA, short chain fatty acids.
