# Supplementary material for: Isolated White Lupin Proteins Beneficially Modulate the Intestinal Microbiota Composition in Rats
Source: Nutrients. 2025 Jan 31;17(3):551. doi: 10.3390/nu17030551 (PMC11820050; doi:10.3390/nu17030551)
Supplement: Supplementary file 1 [file nutrients-17-00551-s001.zip › nutrients-3397747-supplementary.pdf]

ANCOM statistical results

	W
icutes;c__Bacilli;o__Lactobacillales;f__Lactobacillaceae;g__Lactobacillus;s__reuteri	180
icutes;c__Bacilli;o__Lactobacillales;f__Streptococcaceae;g__Lactococcus;s__	173

Download table as TSV

Percentile abundances of features by group

	0.0	25.0	50.0	75.0	100.0	0.0	25.0	50.0	75.0	100.0	0.0	25.0	50.0	75.0	100.0
	Casein	Casein	Casein	Casein	Casein	Lactalbumin	Lactalbumin	Lactalbumin	Lactalbumin	Lactalbumin	Lupin prot. isol.	Lupin prot. isol.	Lupin prot. isol.	Lupin prot. isol.	Lupin prot. isol.
__Lactobacillus;s__reuteri	1.0	1.0	1.0	3.0	24.0	8.0	31.75	149.5	442.25	1645.0	1.0	110.5	169.0	514.0	2706.0
g__Lactococcus;s__	1.0	1.0	1.0	1.0	6.0	1.0	8.25	15.5	26.50	58.0	1.0	1.0	1.0	1.0	1.0

Download table as TSV

Figure S1. ANCOM analysis of the samples from the faeces of rats fed LA, CAS or LPI diets. The clr (centered log ratio) transformed OTU table at the species level that was modified to adjust 0 values to 1 was used. The W value represents the number of times of the null hypothesis (the average abundance of a given species in a group is equal to that in the other group) was rejected for a given species. Only species with reject null-hypothesis >95% are labelled.

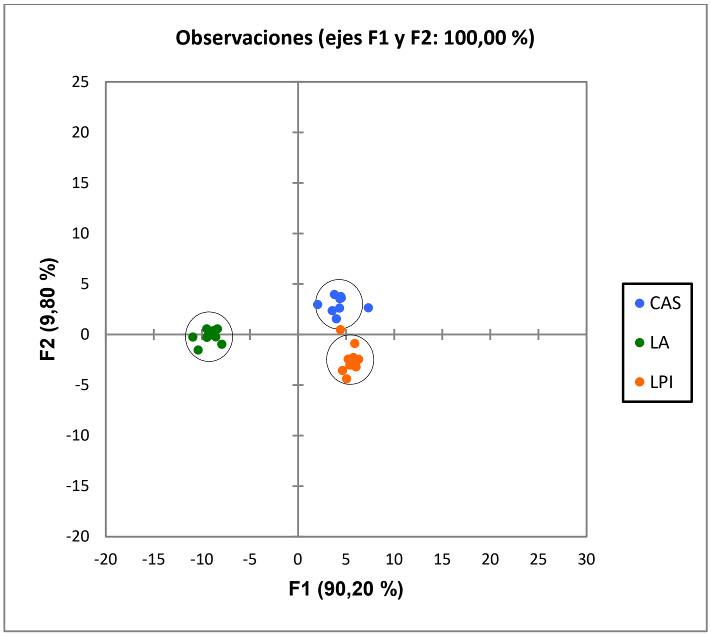

p-values for Fisher distances:			
	CAS	LA	LPI
CAS	1	0,004	0,283
LA	0,004	1	0,003
LPI	0,283	0,003	1

Figure S2. Discriminant Analysis of PICRUSt predicted functions after SIMPER (50% dissimilarity) analysis of the intestinal microbiota bacterial groups analysed by Illumina sequencing; LA, lactalbumin; CAS, casein; LPI, lupin proteins isolate.

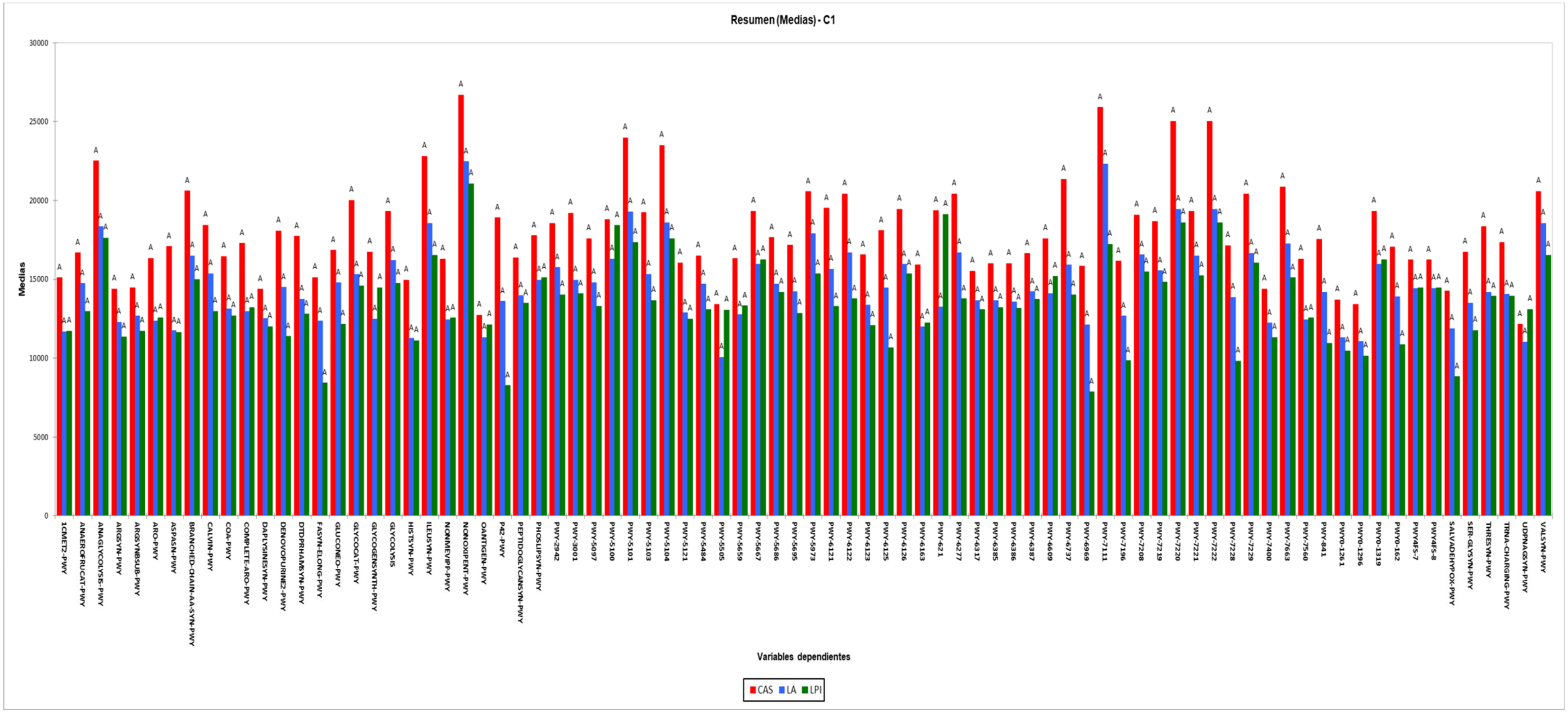

Figure S3. ANOVA of PICRUSt functional analysis after SIMPER (50% dissimilarity) analysis, using the default parameters (LDA score = 2).

No significant differences
